# Risk Factors of Recurrent Ectopic Pregnancy in Patients Treated With *in vitro* Fertilization Cycles: A Matched Case-Control Study

**DOI:** 10.3389/fendo.2020.552117

**Published:** 2020-09-18

**Authors:** Yu Tan, Zhi-qin Bu, Hao Shi, Hui Song, Yi-le Zhang

**Affiliations:** ^1^Reproductive Medicine Center, First Affiliated Hospital of Zhengzhou University, Zhengzhou, China; ^2^Henan Key Laboratory of Reproduction and Genetics, First Affiliated Hospital of Zhengzhou University, Zhengzhou, China

**Keywords:** case-control study, assisted reproductive technology, *in vitro* fertilization, recurrent ectopic pregnancy, risk factors

## Abstract

**Objective:** To study the risk factors for recurrent ectopic pregnancy (REP) in patients undergoing *in vitro* fertilization (IVF).

**Methods:** This was a 1:4 matched case-control study that enrolled 227 REP patients and 908 matched intrauterine pregnancy (IUP) patients from the assisted reproductive technology (ART) center of the First Affiliated Hospital of Zhengzhou University from January 2012 to November 2019. Univariate analysis was carried out between the two groups for the occurrence of REP. Multivariate logistic regression analysis was used to explore the risk factors of REP after IVF.

**Results:** The results of univariate analysis showed that there were significant differences in previous treatment of EP, stage of embryo and the number of embryos transferred between the two groups (all *P* < 0.05). The other factors did not have a significant effect on the probability of developing REP. Multivariate logistic regression analysis showed that after adjusting for confounders, previous treatment of EP, type of embryos transferred and stage of embryo were related to the occurrence of REP (all *P* < 0.05).

**Conclusion:** Conservative treatment, frozen-thawed embryo transfer and cleavage embryo transfer were independent risk factors for REP after ART treatment.

## Introduction

Ectopic pregnancy (EP) accounts for ~2% of all pregnancies. It is a common life-threatening emergency and an important cause of maternal morbidity and mortality, as well as fetal loss (1–3). Although mortality due to EP has declined sharply in recent years, the incidence of EP is still steadily rising at a global level (4, 5). As a long-term complication of EP, recurrent ectopic pregnancy (REP) is also increasing accordingly (5), which has seriously affected the safety and quality of life of childbearing-aged women, and its occurrence and development are related to many factors (6).

With the wide application of assisted reproductive technology (ART) in the field of reproduction, it is of great clinical value to identify the risk factors of REP in infertile patients with a history of EP. If women at risk can be identified in the early stages of ART and be provided with targeted treatment to protect their reproductive ability, they may be able to avoid another occurrence of REP. However, at present, the risk factors related to REP are unclear. In this study, infertile patients with a history of EP were taken as the research objects, and their fertility outcomes were tracked. The patients with REP were taken as the experimental group, and the intrauterine pregnancy (IUP) without REP as the control group. Multivariate logistic regression was used to analyze the risk factors of REP.

## Materials and Methods

### Study Design and Participants

**Figure 1:**
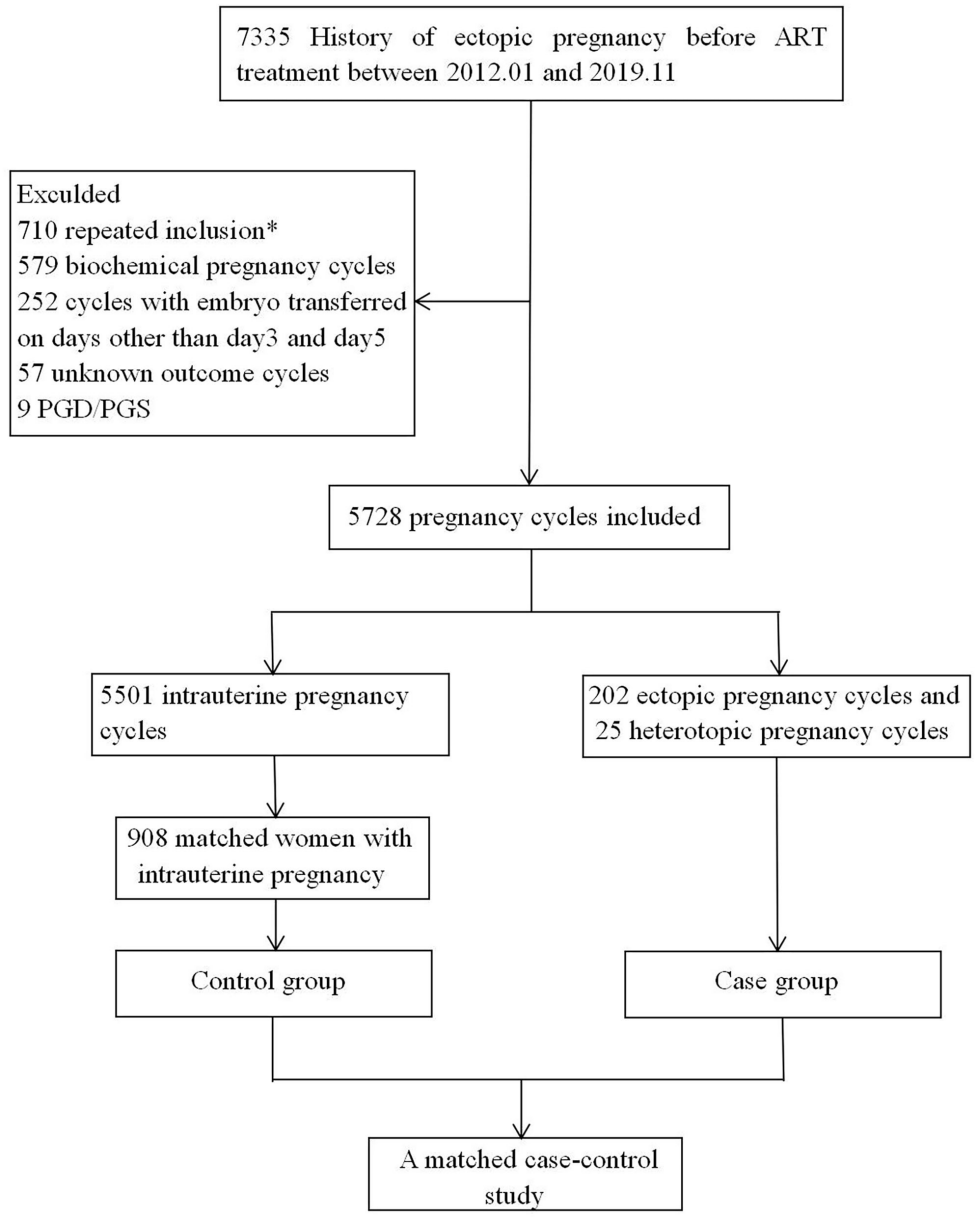
Cycles selection flowchart. *In the case of multiple cycles of clinical pregnancy after ART, bring into the first cycle.

This case-control study was conducted in the ART center of the First Affiliated Hospital of Zhengzhou University from January 2012 to November 2019. The data were extracted from the patients' clinical records. The selection process of the cycles is shown in Figure 1. The inclusion criteria were as follows: (1) a history of EP; (2) ART cycles; (3) generally good physical and mental health (no disability, no mental disorders or mental illness). The exclusion criteria were as follows: (1) repeated inclusion, (2) biochemical pregnancy, (3) cycles with the embryo transferred on days other than day 3 and 5; (4) unknown outcome cycles; (5) PGD/PGS.

We selected patients who completed cycles that resulted in a clinical pregnancy of either REP or IUP. We used age to match the REP women with the IUP group. We used age as a matching factor because previous studies have shown that age is an important risk factor for EP (7, 8). As there are many patients with IUP and few patients with REP, the number disparity was too large to make full use of data, so a proportion of 1:4 was adopted. For each REP patient, four IUP women matched for age were chosen as the control case. The sample sizes were 227 and 908 in the REP and IUP groups, with a ratio of 1:4.

### Definition of Clinical Outcomes

Biochemical pregnancy was defined as a pregnancy diagnosed only by the detection of beta hCG in the serum or urine. Clinical pregnancy was defined as in the fifth week after the embryo transfer, on ultrasound, an intrauterine gestational sac was observed with a cardiac beat. An intrauterine pregnancy was defined as a pregnancy with one or more ultrasound-confirmed gestational sacs in the uterus. Ectopic pregnancy was defined as the gestational sac outside the uterine cavity as observed by ultrasound. Heterotopic pregnancy was defined as the coexistence of an intrauterine sac and an ectopic pregnancy.

### Risk Factor Selection

Based on previously published articles, the following risk factors were included (9, 10): age; body mass index (BMI); infertility duration; infertility factors (tubal factor, ovulation disorders, endometriosis, polycystic ovary syndrome, uterine dysplasia, uterine leiomyoma, chromosome abnormalities and unexplained infertility); gravidity; miscarriages; previous EP; treatment of last EP; type of ART; type of embryo transferred; stage of embryo; FET protocols; number of embryos transferred.

### Treatment Protocol

Patients with fresh embryo transfer were treated with gonadotropin releasing hormone (GnRH) agonists to prevent a premature LH surge (11, 12), and injectable gonadotropins were administered to stimulate follicle growth. We regularly monitored follicle growth by transvaginal ultrasound and the serum estradiol, progesterone and LH levels during the cycle. When at least one follicle had a mean diameter of more than 18 mm, Aizer 250 μg (Merck Serono, Italy) or hCG (Zhuhai Lizhu Medicine) 2,000 IU was given. Ova were collected 36 h later, and subsequent fresh embryo transfers were performed on day 3 or 5. Progesterone (60 mg) was injected intramuscularly on the day of oocyte retrieval. Progesterone gel (Xenotong, Merck Sherano, Switzerland) and oral dydrogesterone [10 mg [Duphaston]; Solvay Pharmaceuticals B.V., Veenendaal, The Netherlands] were given vaginally from the day of embryo transfer for luteal phase support.

The FET protocols in our center were mainly divided into natural cycles and artificial cycles based on the regularity of the menstrual cycle. For natural cycles, patients were allocated to undergo ultrasonic evaluation starting from day 8–9 of the menstrual cycle. The endometrial thickness and mean diameter of the dominant follicle were examined by the same physician. When the diameter of the dominant follicle was 16–20 mm, a blood sample was obtained to determine the progesterone and LH levels.

Thawing and transferring were performed 3 days after ovulation. Intramuscular (im) progesterone (40 mg) starting on the day of ovulation and oral dydrogesterone (20 mg) starting on the embryo transfer day were used for luteal support.

For artificial cycles, patients began oral estradiol [2 mg [Progynova]; Bayer, Leverkusen, Germany] twice a day on cycle day 3. This dose was adjusted based on the endometrial thickness every 4 days. After 12–14 days, an ultrasound was performed and a serum progesterone level was determined. If no leading follicle was present, progesterone (60 mg im) and oral dydrogesterone [10 mg [this dose was changed to 20 mg 2 days later]] would be added to the regimen. Embryo transfer was performed 3 days later.

All of the patients were followed up and hCG biochemical pregnancy tests were performed 2 weeks after the embryo transfer procedure. When the serum hCG was >50 IU/L, the luteal support was continued. Transvaginal ultrasound was performed 5 weeks after embryo transfer.

### Statistical Analysis

SPSS21.0 software was used for the statistical analysis. The general data of the patients were primarily expressed as %; only BMI and infertility duration were expressed by mean ± standard deviation (x ± s). Differences in BMI and infertility duration were analyzed using *t*-tests. Grouping variables were analyzed with chi-square tests. To explore the risk factors of REP, a multivariable logistic regression analysis was used to adjust for potential confounders and to calculate the adjusted odds ratio (AOR). The statistical significance was established at *P* < 0.05.

## Results

### Basic Characteristics of the Patients and a History of EP

A total of 1,135 patients, including 227 REP patients and 908 matched IUP patients, were enrolled in this research. In the REP group, 69% had only one previous episode of EP, while 31% of patients had a history of ≥ 2 ectopic pregnancies. The basic characteristics of the two groups are shown in Table 1. On account of the matching criteria used in our study, there was no significant difference in age between the groups. At the same time, there were no differences in BMI, infertility duration, infertility factors, gravidity, miscarriages or previous EP (all *P* > 0.05). It is worth noting that there was a significant difference in previous treatments for EP between the two groups (*P* < 0.05).

**Table 1:** Univariate analysis of risk factors related to basic characteristics of patients.

**Variables**	**IUP, n (%)**	**REP, n (%)**	***χ^2^*/*t***	***P*-value**
Total	908	227		
Age			0.000	1.000
<30	424 (80)	106 (20)		
30–35	380 (80)	95 (20)		
>35	104 (80)	26 (20)		
Body mass index (kg/m^2^)	22.75 ± 3.06	22.66 ± 2.91	0.420	0.675
Infertility duration (years)	2.65 ± 2.51	2.52 ± 2.16	0.756	0.450
Infertility factors			4.747	0.093
Tubal factor	645 (81.23)	149 (18.77)		
Compound factors^*^	199 (75.38)	65 (24.62)		
Others^**^	64 (83.12)	13 (16.88)		
Gravidity			1.780	0.411
1	323 (78.78)	87 (21.22)		
2	304 (79.17)	80 (20.83)		
≥3	281 (82.40)	60 (17.60)		
Miscarriages			1.704	0.427
0	511 (80.09)	127 (19.91)		
1	242 (78.06)	68 (21.94)		
≥2	155 (82.89)	32 (17.11)		
Previous EP			3.485	0.062
1	618 (78.53)	169 (21.47)		
≥2	290 (83.33)	58 (16.67)		
Treatment of last EP			6.883	0.032
Salpingectomy	443 (83.27)	89 (16.73)		
Conservative operation	391 (77.43)	114 (22.57)		
Methotrexate	74 (75.51)	24 (24.49)		

* *Including tubal factor*.

** *Including ovulation disorders, endometriosis, polycystic ovary syndrome, uterine dysplasia, uterine leiomyoma, chromosome abnormalities, and unexplained infertility*.

### Risk Factors Associated With ART Cycles

A total of 661 fresh embryo transfer cycles and 474 frozen-thawed embryo transfer (FET) cycles were included as shown in Table 2. The REP rate of fresh embryo transfer was lower than that of the FET, although there was no significant difference between the two groups. Moreover, the type of ART and FET protocols showed no significant correlation with the probability of developing REP (*P* > 0.05). Compared with IUP, the frequency of REP in the cleavage stage was higher, while the frequency of REP in the blastocyst stage was lower. The outcome of ART also depended on the number of embryos transferred. REP was more likely to occur when two or more embryos were transferred in each cycle.

**Table 2:** Univariate analysis of risk factors associated with fresh and frozen cycles.

**Variables**	**IUP, n (%)**	**REP, n (%)**	***χ^2^***	***P*-value**
Total	908	227		
Type of ART			0.063	0.802
IVF	821 (80.10)	204 (19.90)		
ICSI	87 (79.09)	23 (20.91)		
Type of transfer			3.370	0.066
Fresh embryo	541 (81.85)	120 (18.15)		
Thawed embryo	367 (77.43)	107 (22.57)		
Stage of embryo			29.013	0.000
Cleavage stage	641 (76.22)	200 (23.78)		
Blastocyst stage	267 (90.82)	27 (9.18)		
FET protocols			0.026	0.872
Natural cycle	130 (77.84)	37 (22.16)		
Artificial cycle	237 (77.20)	70 (22.80)		
No. of embryos transferred			16.933	0.000
1	200 (89.29)	24 (10.71)		
2	640 (78.34)	177 (21.66)		
3	68 (72.34)	26 (27.66)		

### Multivariable Analysis of Risk Factors for REP

To evaluate the effect of these risk factors on the occurrence of REP after IVF, we included infertility factors, previous EP, previous treatment of EP, type of embryo transferred, stage of the embryo and the number of embryos transferred for multivariate logistic regression analysis, as shown in Table 3. In women with a history of EP, the infertility factors, previous EP, and the number of embryos transferred (all *P* > 0.05) had no correlation with the occurrence of REP. Compared with salpingectomy, prior treatment of EP with a conservative operation (AOR = 1.484, 95% CI = 1.078–2.042) and methotrexate (AOR = 1.729, 95% CI = 1.007–2.967) increased the risk of REP. In addition, the risk of REP in fresh embryo transfer cycles was lower than that in FET cycles (AOR = 1.651, 95% CI = 1.203–2.265). Moreover, blastocyst transfer (AOR = 0.275, 95% CI = 0.158–0.479) also had a lower incidence of REP than cleavage transfer.

**Table 3:** Risk factors associated with REP by logistic regression analysis.

**Factor**	**Adjusted OR (95% CI)**	***P*-value**
Infertility factors		
Tubal factor	REF	
Compound factors	1.320 (0.936–1.861)	0.113
Others	0.730 (0.378–1.412)	0.350
Previous EP		
1	REF	
≥2	0.738 (0.525–1.037)	0.080
Treatment of last EP		
Salpingectomy	REF	
Conservative operation	1.484 (1.078–2.042)	0.015
Methotrexate	1.729 (1.007–2.967)	0.047
Type of transfer		
Fresh embryo	REF	
Thawed embryo	1.651 (1.203–2.265)	0.002
Stage of embryo		
Cleavage stage	REF	
Blastocyst stage	0.275 (0.158–0.479)	0.000
No. of embryos transferred		
1	REF	
2	0.981 (0.551–1.746)	0.947
3	1.115 (0.527–2.360)	0.775

*Included variables: infertility factors, previous EP, previous treatment of EP, type of embryo transferred, stage of the embryo, and the number of embryos transferred*.

*OR, odds ratio; CI, confidence interval; REF, reference*.

## Discussion

Ectopic pregnancy (EP) is a common cause of acute abdomen in obstetrics and gynecology, and it is also one of the important causes of maternal death. While ART meets people's fertility needs, it has a risk of EP, which can easily increase the incidence of REP (13, 14). Therefore, researchers are committed to exploring the risk factors and methods of preventing EP during ART treatment cycles.

### Previous History of EP and REP

According to the statistics, the recurrence rate of EP ranges from 10 to 27% in the general population (5). Patients with a previous history of EP are at a higher risk of REP (15): about a third of patients would develop EP again after the first EP. The main risk factor for EP is fallopian tube damage. Patients with a previous history of pelvic infection, especially those who underwent a conservative operation or tubal microsurgery for a tubal pregnancy, have a higher incidence of EP after assisted pregnancy (16, 17). The subjects in this study were all patients with a history of EP. The recurrence rate of EP in patients undergoing ART and its correlation with previous treatments of EP were investigated in this study.

The management of EP includes expectant behavior, conservative drug treatment (methotrexate) and surgical treatment. Surgical treatment could be divided into conservative operations and radical resection. No matter what kind of treatment is used in patients with EP, the pathological changes of the fallopian tubes might persist, or the fallopian tubes on the affected side might adhere again, resulting in secondary infertility.

When applying general drug conservative treatment, drugs are used to interfere with the synthesis of DNA and RNA of the embryonic cells, making it difficult for them to grow and stopping the development of the embryo, but the damage the pregnancy products inflict on the fallopian tubes and any subsequent tubal inflammation cannot be effectively removed. Zhang Anhong (18) found that among 156 patients with tubal pregnancy, the recurrence rate of tubal pregnancy in the drug conservative treatment group (16.67%) was significantly higher than that in the diseased side salpingectomy group (6.94%). Our research came to a similar conclusion, that previous treatment of EP with methotrexate (24.49%) increased the risk of REP after ART treatment compared with salpingectomy (16.73%).

The surgical treatment of tubal pregnancy includes tubal sparing focus clearance (conservative operation) and salpingectomy on the affected side. Some studies have suggested that previous treatment of EP with a conservative operation had a comparable risk of REP as salpingectomy (19, 20). The results of this study showed that comparing with salpingectomy, the risk of REP after a conservative operation was increased. Some previous reports are consistent with our findings (21, 22). In addition, our data also showed that there was no correlation between REP and the number of previous EPs. In contrast, a previous study reported that the risk of REP was increased with a history of a higher number of previous EPs (23).

### ART Cycles and REP

Many previous studies have evaluated the incidence of EP after ART, but there have been few reports on a specific population with a history of EP. Additionally, it was not clear whether there was a difference in the recurrence rate of EP between fresh and frozen cycles. The subjects of this research were all patients with a history of EP. The results showed that the risk of REP in fresh cycles was lower than that in frozen cycles. Pan Le Le's research results showed that the recurrence rate of EP in fresh cycles was 4.99%, which was lower than that of frozen cycles (11.03%), and the difference was statistically significant (24). This was consistent with the results of our study. In the current research, we did not find that different types of ART were related to REP, and the effect of IVF/ICSI on the recurrence of EP still needs to be determined by further research.

Although the effects of blastocyst embryo vs. cleavage embryo transfer on the incidence of EP remain controversial, most studies have shown that blastocyst embryo transfer is beneficial for reducing the incidence of EP (25, 26). This research also supported this view. Blastocyst culture is helpful to screen for embryos with good developmental potential and to improve the embryo implantation rate. Moreover, compared with cleavage embryos, the intrauterine transfer of blastocysts is closer to the physiological state, and the development of the endometrium and embryos tends to be synchronized, which shortens the interval between further development and implantation after the embryo is transferred into the uterine cavity. At the same time, due to the increased progesterone level in the body, inhibition of uterine myometrium contractions could reduce the chance of embryo migration and is more conducive to implantation. In addition, the blastocyst volume is larger, compared with cleavage embryos, making it more difficult for it to migrate to the fallopian tube (27, 28).

As for the number of embryos transferred, theoretically, the incidence of EP would increase with an increased number of transferred embryos, and some studies such as Perkins et al. (29) have come to a similar conclusion. However, the results of some previous researchers mentioned that more than one transfer could not be considered a risk factor for EP (9, 30). The data from our study also indicated that there was no significant difference in the recurrence of EP with different numbers of transferred embryos.

### Strengths and Limitations

The main strength of our study was that we are the first to investigate the risk factors of REP after ART in patients with a history of EP. In addition, this was an original study. However, our study also had several limitations due to its retrospective design and a single medical center. In addition, this research is only a preliminary discussion, due to the small sample size and collection time, and there is an inevitable bias. It is necessary to expand the sample size for further research. Therefore, the conclusions needed to be interpreted carefully.

In summary, conservative treatment, FET and cleavage embryo transfer were independent risk factors for REP after ART treatment. Patients with conservative treatment should be on guard against the occurrence of REP. This suggests that salpingectomy on the affected side should be selected when there is a surgical indication for an EP. In addition, during the course of ART treatment, fresh cycles or blastocyst embryo transfer should be selectively performed for populations at high risk of REP.

## Data Availability Statement

All datasets generated for this study are included in the article.

## Ethics Statement

All study methods were approved by the Ethics Committee of the First Affiliated Hospital of Zhengzhou University (Scientific Research-2020-KY-256). All participants provided written informed consent. All studies were conducted in accordance with the relevant guidelines and regulations.

## Author Contributions

All authors listed have made a substantial, direct and intellectual contribution to the work, and approved it for publication.

## Conflict of Interest

The authors declare that the research was conducted in the absence of any commercial or financial relationships that could be construed as a potential conflict of interest.

## References

[1] Ononuju CN Ogbe AE Changkat LL Okwaraoha BO Chinaka UE. Ectopic pregnancy in Dalhatu Araf specialist hospital lafia nigeria - a 5-year review. Niger Postgrad Med J. (2019) 26:235–8. 10.4103/npmj.npmj_105_19 31621664

[2] Bozkaya G Karaca I Fenercioglu O Yildirim KS Bilgili S Uzuncan N. Evaluation of maternal serum ischemia modified albumin and total antioxidant status in ectopic pregnancy. J Matern Fetal Neonatal Med. (2019) 32:2003–8. 10.1080/14767058.2017.1422718 29284337

[3] Brady Paula C. New evidence to guide ectopic pregnancy diagnosis and management. Obstet Gynecol Surv. (2017) 72:618–25. 10.1097/OGX.0000000000000492 29059454

[4] Shobeiri F Tehranian N Nazari M. Trend of ectopic pregnancy and its main determinants in Hamadan province, Iran (2000-2010). BMC Res Notes. (2014) 7:733. 10.1186/1756-0500-7-733 25326269 PMC4283128

[5] Butts S Sammel M Hummela A Chittams J Barnhart K. Risk factors and cinical features of recurrent ectopic pregnancy: a case control study. Fertil Steril. (2003) 80:1340–4. 10.1016/S0015-0282(03)02206-4 14667866

[6] Levin G Dior UP Shushan A Gilad R Benshushan A Rottenstreich A. Risk factors for recurrent ectopic pregnancy following single-dose methotrexate treatment. Eur J Contracept Reprod Health Care. (2019) 24:294–8. 10.1080/13625187.2019.1625324 31204856

[7] Hurrell A Reeba O Funlayo O. Recurrent ectopic pregnancy as a unique clinical sub group: a case control study. Springerplus. (2016) 5:265. 10.1186/s40064-016-1798-0 27006874 PMC4775712

[8] Al-Turki Haifa A. Ectopic pregnancy. Prevalence and risk factors in women attending a tertiary care hospital in Saudi Arabia. Saudi Med J. (2012) 33:875–8. Available online at: https://pubmed.ncbi.nlm.nih.gov/22886121/22886121

[9] Bu Z Xiong Y Wang K Sun Y. Risk factors for ectopic pregnancy in assisted reproductive technology: a 6-year, single-center study. Fertil Steril. (2016) 106:90–4. 10.1016/j.fertnstert.2016.02.035 27001382

[10] Liu X Qu P Bai H Shi W Shi J. Endometrial thickness as a predictor of ectopic pregnancy in 1125 *in vitro* fertilization-embryo transfer cycles: a matched case-control study. Arch Gynecol Obstet. (2019) 300:1797–803. 10.1007/s00404-019-05353-z 31720777

[11] Hu L Bu Z Guo Y Su Y Zhai J Sun Y. Comparison of different ovarian hyperstimulation protocols efficacy in poor ovarian responders according to the Bologna criteria. Int J Clin Exp Med. (2014) 7:1128–34. Available online at: https://www.ncbi.nlm.nih.gov/pmc/articles/PMC4057873/24955194 PMC4057873

[12] Kong H Hu L Nie L Yu X Dai W Li J. A multi-center, randomized controlled clinical trial of the application of a shortened protocol of long-acting Triptorelin down-regulated prior to IVF / ICSI among patients with endometriosis: a protocol. Reprod Health. (2018) 15:213. 10.1186/s12978-018-0639-8 30572916 PMC6302481

[13] Chang HJ Suh CS. Ectopic pregnancy after assisted reproductive technology: what are the risk factors? Curr Opin Obstet Gynecol. (2010) 22:202–7. 10.1097/GCO.0b013e32833848fd 20216415

[14] Refaat B Dalton E Ledger WL. Ectopic pregnancy secondary to *in vitro* fertilisation-embryo transfer: pathogenic mechanisms and management strategies. Reprod Biol Endocrinol. (2015) 13:30. 10.1186/s12958-015-0025-0 25884617 PMC4403912

[15] Yoder N Tat R Martin JR. Abdominal ectopic pregnancy after *in vitro* fertilization and single embryo transfer: a case report and systematic review. Reprod Biol Endoerinol. (2016) 14:69. 10.1186/s12958-016-0201-x 27760569 PMC5070159

[16] Lawani OL Anozie OB Ezeonu PO. Ectopic pregnancy: a lifethreatening gynecological emergency. Int J Womens Health. (2013) 5:515–21. 10.2147/IJWH.S49672 23983494 PMC3751381

[17] Bhattacharya S McLernon DJ Lee AJ Bhattacharya S. Reproductive outcomes following ectopic pregnancy: register-based retrospective cohort study. PLoS Med. (2012) 9:e1001243. 10.1371/journal.pmed.1001243 22723747 PMC3378618

[18] Zhang A Analysis of reproductive function of 156 cases of tubal pregnancy after treatment. Jiangsu Med. (2014) 40:464–5. 10.19460/j.cnki.0253-3685.2014.04.034

[19] Kostrzewa M Zyla M Litwińska E Kolasa-Zwierzchowska D Szpakowski A Stachowiak G. Salpingotomy vs salpingectomy–a comparison of women's fertility after surgical treatment of tubal ectopic pregnancy during a 24-month follow-up study. Ginekol Pol. (2013) 84:1030–35. 10.17772/gp/1675 24505950

[20] Xu Z Yan L Liu W Xu X Li M Ding L. Effect of treatment of a previous ectopic pregnancy on *in vitro* fertilization-intracytoplasmic sperm injection outcomes: a retrospective cohort study. Fertil Steril. (2015) 104:1446–51. 10.1016/j.fertnstert.2015.08.034 26409152

[21] Zhang D Shi W Li C Yuan JJ Xia W Xue RH. Risk factors for recurrent ectopic pregnancy: a case-control study. BJOG. (2016) 123:82–9. 10.1111/1471-0528.14011 27627605

[22] Ellaithy M Asiri M Rateb A Altraigey A Abdallah K. Prediction of recurrent ectopic pregnancy: a five-year follow-up cohort study. Eur J Obstet Gynecol Reprod Biol. (2018) 225:70–8. 10.1016/j.ejogrb.2018.04.007 29679814

[23] Irani M Robles A Gunnala V Spandorfer SD. Unilateral salpingectomy and methotrexate are associated with a similar recurrence rate of ectopic pregnancy in patients undergoing *in vitro* fertilization. J Minim Invasive Gynecol. (2017) 24:777–82. 10.1016/j.jmig.2017.03.002 28285056

[24] Pan L Yang J Xiao G Li N Jin J Song J. Analysis of the incidence and risk factors of ectopic pregnancy in fresh and frozen-thawed embryo transfer cycles. Reprod Contracept. (2015) 35:606–11. 29254433

[25] Du T Chen H Fu R Chen Q Wang Y Mol BW. Comparison of ectopic pregnancy risk among transfers of embryos vitrified on day 3, day 5, and day 6. Fertil Steril. (2017) 108:108–16. 10.1016/j.fertnstert.2017.05.027 28602476

[26] Santos-Ribeiro S Tournaye H Polyzos NP. Trends in ectopic pregnancy rates following assisted reproductive technologies in the UK: a 12-year nationwide analysis including 160 000 pregnancies. Hum Reprod. (2016) 31:393–402. 10.1093/humrep/dev315 26724796

[27] Mangalraj AM Muthumar K Aleyamma T Kamath MS George K. Blastocyst stage transfer vs cleavage stage embryo transfer. J Hum Repord Sci. (2009) 2:23–6. 10.4103/0974-1208.51339 19562070 PMC2700692

[28] Li Z Sullivan EA Chapman M Farquhar C Wang YA. Risk of ectopic pregnancy lowest with transfer of single frozen blastocyst. Hum Reprod. (2015) 30:2048–54. 10.1093/humrep/dev168 26202917

[29] Perkins KM Boulet SL Kissin DM Jamieson DJ National ART Surveillance (NASS) Group. Risk of ectopic pregnancy associated with assisted reproductive technology in the United States, 2001-2011. Obstet Gynecol. (2015) 125:70–8. 10.1097/AOG.0000000000000584 25560107 PMC4315158

[30] Gelbaya TA Tsoumpou I Nardo LG. The likelihood of live birth and multiple birth after single versus double embryo transfer at the cleavage stage: a systematic review and meta-analysis. Fertil Steril. (2010) 94:936–45. 10.1016/j.fertnstert.2009.04.003 19446809

